# Pregnancy in myasthenia gravis: a retrospective analysis of maternal and neonatal outcome from a large tertiary care centre in Germany

**DOI:** 10.1007/s00404-024-07436-y

**Published:** 2024-03-16

**Authors:** Jakob Draxler, Andreas Meisel, Frauke Stascheit, Maike Stein, Lea Gerischer, Philipp Mergenthaler, Meret Herdick, Paolo Doksani, Sophie Lehnerer, Stefan Verlohren, Sarah Hoffmann

**Affiliations:** 1 Department of Neurology, Neuroscience Clinical Research Center (NCRC) and Integrated Myasthenia Gravis Center, Charité - Universitätsmedizin, Corporate Member of Freie Universität Berlin and Humboldt-Universität zu Berlin, Charitéplatz 1, 10117 Berlin, Germany; 2 Berlin Institute of Health at Charité – Universitätsmedizin Berlin, Charitéplatz 1, 10117 Berlin, Germany; 3 Department of Obstetrics, Charité - Universitätsmedizin, Corporate Member of Freie Universität Berlin and Humboldt-Universität zu Berlin, Charitéplatz 1, 10117 Berlin, Germany; 4 Center for Stroke Research Berlin, Charité – Universitätsmedizin Berlin, Charitéplatz 1, 10117 Berlin, Germany; 5 Radcliffe Department of Medicine, University of Oxford, Oxford, UK

**Keywords:** Exacerbation, Delivery, Transient neonatal myasthenia gravis, FARAD, Arthrogryposis multiplex congenita

## Abstract

**Purpose:**

Myasthenia gravis (MG) is a rare, potentially life-threatening autoimmune disease with fluctuating muscle weakness frequently affecting women of childbearing age. MG can affect maternal as well as neonatal outcome with risk of worsening of myasthenic symptoms in the mothers and risk of transient neonatal myasthenia gravis (TNMG) and arthrogryposis multiplex congenita (AMC) or foetal acetylcholine receptor antibody-associated disorders (FARAD) in the neonates.

**Methods:**

Retrospective analysis of maternal and neonatal outcome in a cohort of pregnant MG patients treated at a tertiary care centre in Germany.

**Results:**

Overall, 66 pregnancies were analysed. During 40 (63%) pregnancies, women experienced a worsening of myasthenic symptoms, of whom 10 patients (15.7%) needed acute therapy with IVIg or plasma exchange. There was no case of myasthenic crisis. Rate of caesarean section was comparable to the overall C-section rate at our centre (38% vs. 40%). However, there was a slightly higher rate for operative vaginal delivery (15% vs. 10%) as potential indicator for fatiguing striated musculature in MG patients during the expulsion stage. Rate of TNMG as well as AMC was 3% (two cases each).

**Conclusions:**

Maternal and neonatal outcome in our cohort was favourable with a low rate of myasthenic exacerbations requiring acute therapies and a low rate of TNMG and AMC/FARAD. Our data might help neurologists and obstetricians to advice MG patients with desire to have children.

## What does this study add to the clinical work

**Table:** 

Our study shows a favorable maternal and neonatal outcome in pregnancies of MG patients with a low rate of myasthenic exacerbations requiring acute therapies in the mothers and a low rate of transient neonatal myasthenia gravis (TNMG) and arthrogryposis multiplex congenita (AMC) or fetal acetylcholine receptor antibody-associated disorders (FARAD) in the neonates. These findings might help obstetricians as well as neurologists to advise and provide informed guidance to MG patients who wish to have children to do so under the safest conditions possible.	

## Introduction

Myasthenia gravis (MG) is an autoimmune disease mediated by autoantibodies directed against postsynaptic antigens of the neuromuscular junction leading to fatigable muscle weakness that can be life-threatening if respiratory muscles are affected [1]. MG is an orphan disease with an estimated incidence rate of 2.1 and prevalence rate of 22.2 per 100.000 patient years in Germany [2]. There is an overall increasing incidence of MG with age, but women often show a bimodal distribution in incidence rates with a first peak between their 2nd and 4th decade of life coinciding with reproductive age [3]. In clinical practice, questions and concerns of female MG patients with regard to family planning and pregnancy are a frequent topic. A study including over 800 female MG patients showed that more than 50% had abstained from having children due to their disease [4]. Knowledge about the course of MG during pregnancy and its potential impact on neonatal outcome is crucial for neurologists and obstetricians in order to advise and provide informed guidance to MG patients who wish to have children to do so under the safest conditions possible.

Pregnancy in MG patients can have consequences for both mother and child. About one-third of pregnant MG patients experience worsening of myasthenic symptoms, with the highest risk during the first trimester and the post-partum period [5–7]. It is a matter of debate whether rates of preterm birth (PTB), small for gestational age (SGA) and caesarean section (C-section) or operative vaginal delivery are increased in MG patients [5, 8]. Reported rates of transient neonatal myasthenia gravis (TNMG) caused by a transplacental transfer of maternal autoantibodies to the fetal blood circulation vary between 10 and 20% and the clinical picture ranges from general hypotonia and poor sucking to respiratory insufficiency [9, 10]. Symptoms of TNMG usually appear within 24 h after birth and resolve within 2–4 weeks. In most cases, no treatment is required but, if necessary, low doses of acetylcholine esterase inhibitors or in very rare cases intravenous immunoglobulins or plasma exchange can be applied [11, 12]. Arthrogryposis multiplex congenita (AMC) and fetal acetylcholine receptor inactivation syndrome (FARIS) or fetal acetylcholine receptor antibody-associated disorders (FARAD) result from a predominant inhibition of the fetal subunit of the acetylcholine receptor during a critical period of fetal muscle development [13–15]. These are rare but feared conditions due to persistent sequela in the child that can only be addressed by supportive treatment.

The present study summarises the experience gained within a large cohort of MG patients from a German-certified integrated myasthenia gravis centre over a 14-year period to estimate maternal and neonatal outcomes for the above-mentioned complications and to provide rational advice to women on family planning.

## Materials and methods

### Study design and patients

This is a retrospective analysis of data collected at the certified Integrated Myasthenia gravis Center (IMZ) Charité—Universitätsmedizin Berlin providing care for more than 2000 MG patients. Pregnant patients with autoimmune MG with repeated consultation at the IMZ between 01/2008 and 05/2022 were included in this analysis. Patients were identified by a local database query using the search terms pregnant, pregnancy, family planning, desire to have children, week of pregnancy, abortion, delivery/birth, caesarean delivery, sectio, peripartal, post-partum, spontaneous vaginal birth. To patients with missing data, a paper CRF was sent by mail with a request for completion of data.

### Assessment

Assessment of maternal parameters and outcome included gravida, parity, abortion rate, age at delivery, disease duration until delivery, antibody status (AchR, MuSK, seronegative), MG-specific medication (pyridostigmine, prednisolone, nonsteroid immunosuppressive therapy [NSIT], intravenous immunoglobulins [IVIg], plasma exchange [PLEX]), history of thymectomy and thymus histopathology [thymus hyperplasia, thymoma, no abnormalities], rate and timing of clinical worsening during pregnancy and post-partum period, mode of delivery, presence of other autoimmune diseases, fetal ultrasound parameters including pulsatility indices of the uterine and umbilical arteries in the 2nd and 3rd trimester. According to the German guidelines, we define myasthenic exacerbations in our clinic if all of the following criteria are fulfilled [16]: 1. Objectively: QMG (quantitative myasthenia gravis) score of ≥ 8 points and a minimum increase of ≥ 5 points from the previous visit, [2] Subjectively: progressive clinical deterioration due to weakness of bulbo-pharyngeal or limb muscles or reduced respiratory function impacting activities of daily living, [3] Period of time: progress of symptoms no longer than 30 days. This means that a “real” myasthenic exacerbation will lead to an escalation therapy with intravenous IVIg or plasma exchange. We refer to symptom aggravation not fulfilling the criteria of exacerbation as “worsening”.

Assessment of neonatal outcome included prematurity, weight at birth, small for gestational age (SGA), APGAR score at 1, 5 and 10 min, rate of TNMG and AMC. Prematurity was defined as delivery before completion of 37 weeks of gestation. Neonates were considered SGA if their birth weight was less than the 10th percentile for gestational age based on the growth curves. We considered each pregnancy as an individual case, also for women with multiple pregnancies. Banner et al. recently published a systematic review on pregnancy in MG and found that previously published literature on this topic chose this methodology and followed this approach in their own case series [5]. Since comparative statistics are not included in our analysis, we also applied this strategy although multiple pregnancies in the same women may have correlated outcomes.

### Statistical analysis

Sociodemographic as well as clinical parameters were assessed with descriptive analysis including absolute and relative frequencies, means and standard deviation. For statistical analyses, patients with missing values on a specific variable were excluded from analysis. Statistics were performed using SPSS (IBM SPSS Statistics, Version 29.0). Tables and graphics were created using Microsoft Excel.

## Results

### Baseline characteristics

A total of 66 pregnancies in 55 MG patients including three twin pregnancies were recorded. Mean maternal age at birth was 32.3 years (SD 4.2). 60 (87%) patients were positive for AChR-ab, one (1.4%) patient was positive for MuSK-ab and eight (11.6%) patients were seronegative. Rate of thymectomy prior to birth was 40 (60%), with 26 (44%) having thymus hyperplasia and 6 (10%) a history of thymoma. The majority of patients 47 (68.1%) were treated with pyridostigmine, 17 (25%) were treated with prednisolone and 20 (29%) with NSIT, 20 (29%) patients did not take MG-specific medication (see Table 1). In 25 (36.2%) pregnancies, another autoimmune disease was present including Hashimoto thyroiditis (*n* = 10,14.5%), M. Basedow (*n* = 3, 4.3%), systemic lupus erythematodes (*n* = 6, 8.7%), antiphospholipid syndrome (*n* = 4, 5.8%), atopic dermatitis (*n* = 4, 5.8%), autoimmune hepatitis (*n* = 2, 2.9%), rheumatoid arthritis (*n* = 2, 2.9%), Evans syndrome (*n* = 1, 1.4%), Crohn´s disease (*n* = 1, 1.4%), multiple sclerosis (*n* = 1, 1.4%) and Sjögren´s syndrome (*n* = 1, 1.4%).

**Table 1:** Baseline characteristics

Characteristic	
Maternal age at delivery, y, mean (SD)	32.3 (4.2)
Gravida, mean (SD)	2.1 (1.8)
Parity, mean (SD)	1.6 (0.9)
Abortion, *n* (%)	17 (29)
Pregnancies with symptom worsening, *n* (%)	40 (63)
Disease duration at delivery, y, mean (SD)	6.0 (6.3)
Antibody status, *n* (%)	
AchR	60 (87)
MuSK	1 (1.4)
Seronegative	8 (11.6)
Medication, *n* (%)	
Pyridostigmine monotherapy	21 (30.4)
Pyridostigmine overall	47 (68.1)
Prednisolone	17 (24.6)
NSIT	20 (29.0)
None	20 (29.0)
Thymectomy prior to birth, *n* (%)	40 (60)
Thymoma	6 (10)
Thymus hyperplasia	26 (44)
Other autoimmune disease	25 (36.2)
Foetal ultrasound	
PI uterine artery (SD)	
2nd trimenon	0.8 (0.4)
3rd trimenon	0.7 (0.2)
PI umbilical artery (SD)	
2nd trimenon	1.0 (0.2)
3rd trimenon	0.9 (0.2)

*AchR* acetylcholine receptor; *IVIg* intravenous immunoglobulins; *MuSK *muscle-specific tyrosine kinase, *MGFA *Myasthenia gravis foundation of America classification; *NSIT *nonsteroid immunosuppressive therapy; PI pulsatility index; *PLEX *plasma exchange

### Course of MG during pregnancy and mode of delivery

**Fig. 1:**
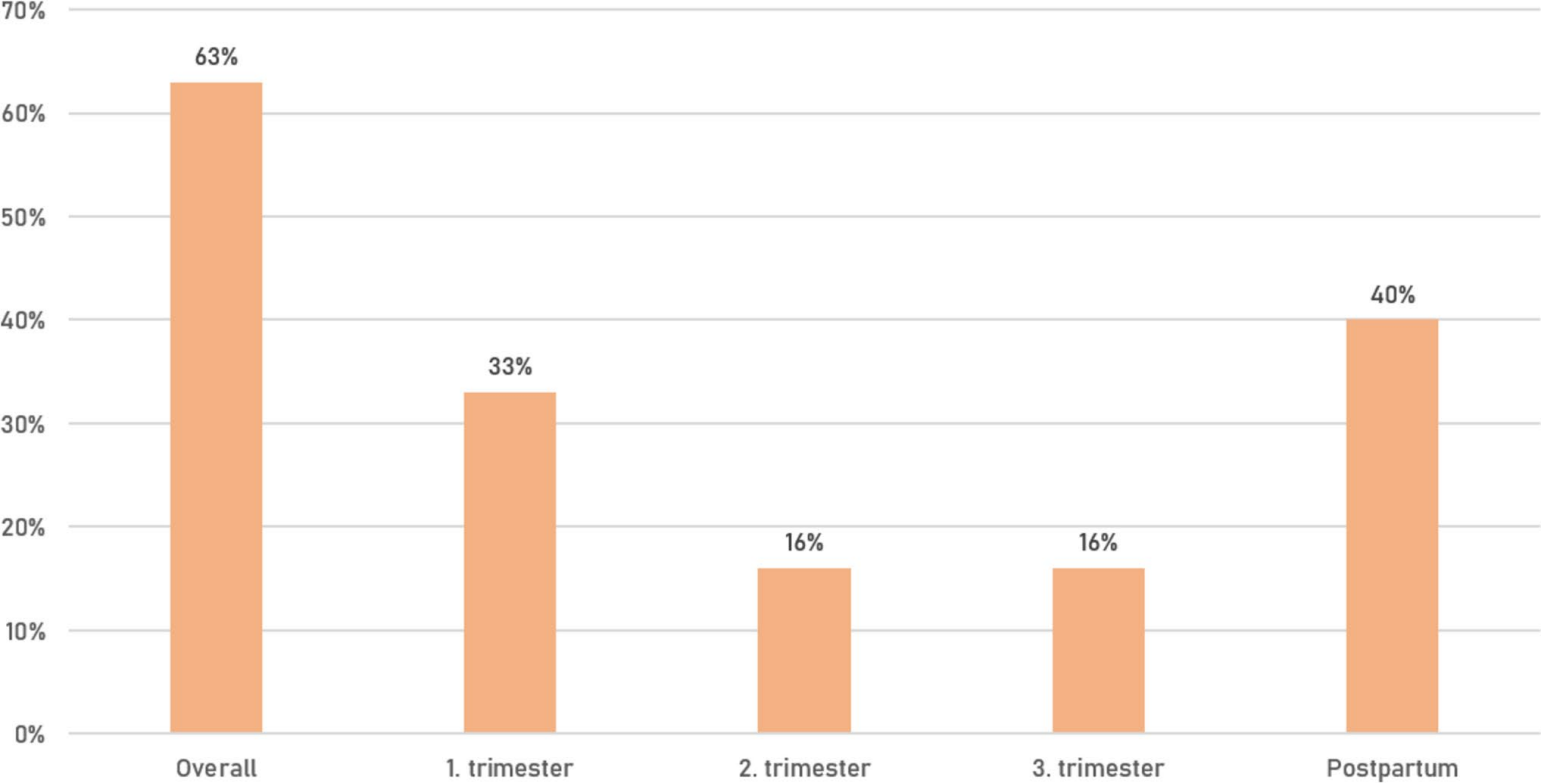
Worsening of myasthenic symptoms per phase of pregnancy

**Fig. 2:**
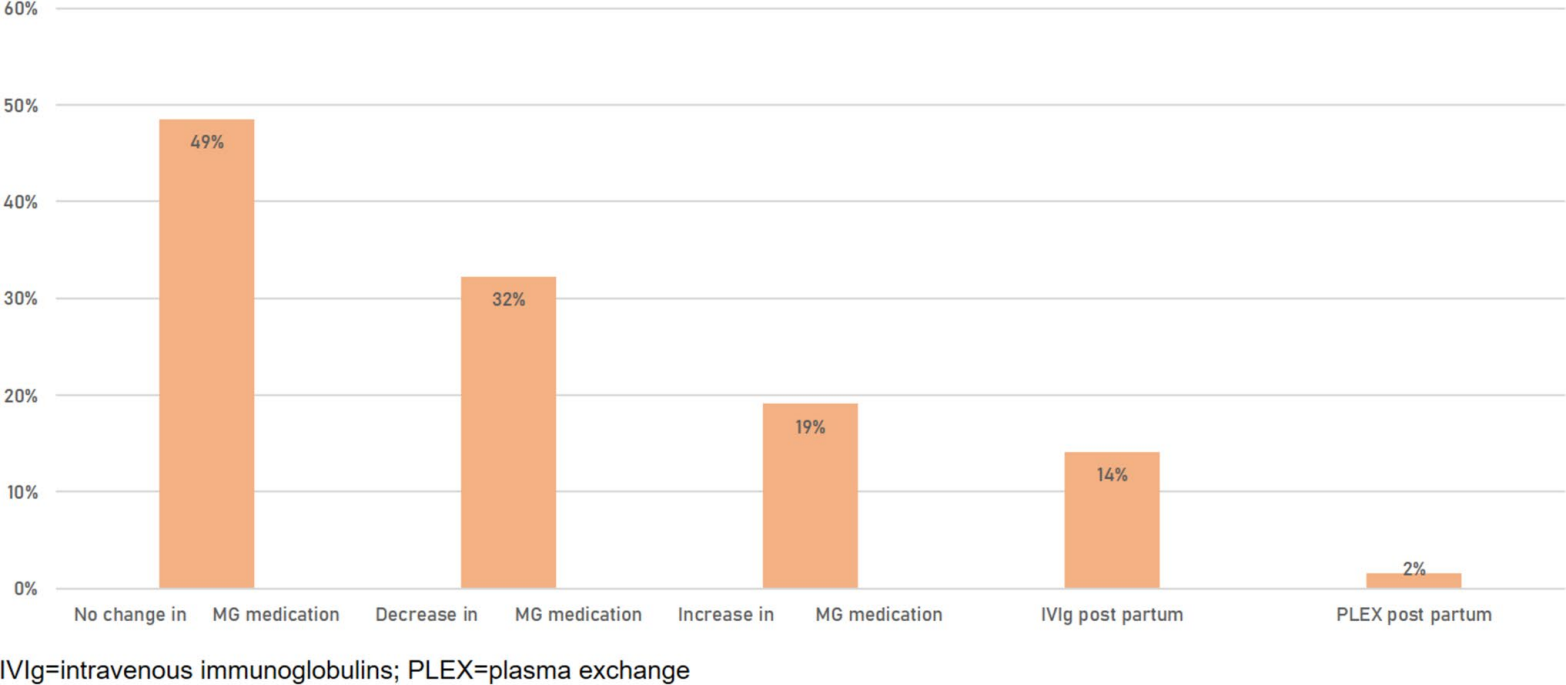
Change of MG-specific medication during pregnancy and post-partum escalation therapies

**Fig. 3:**
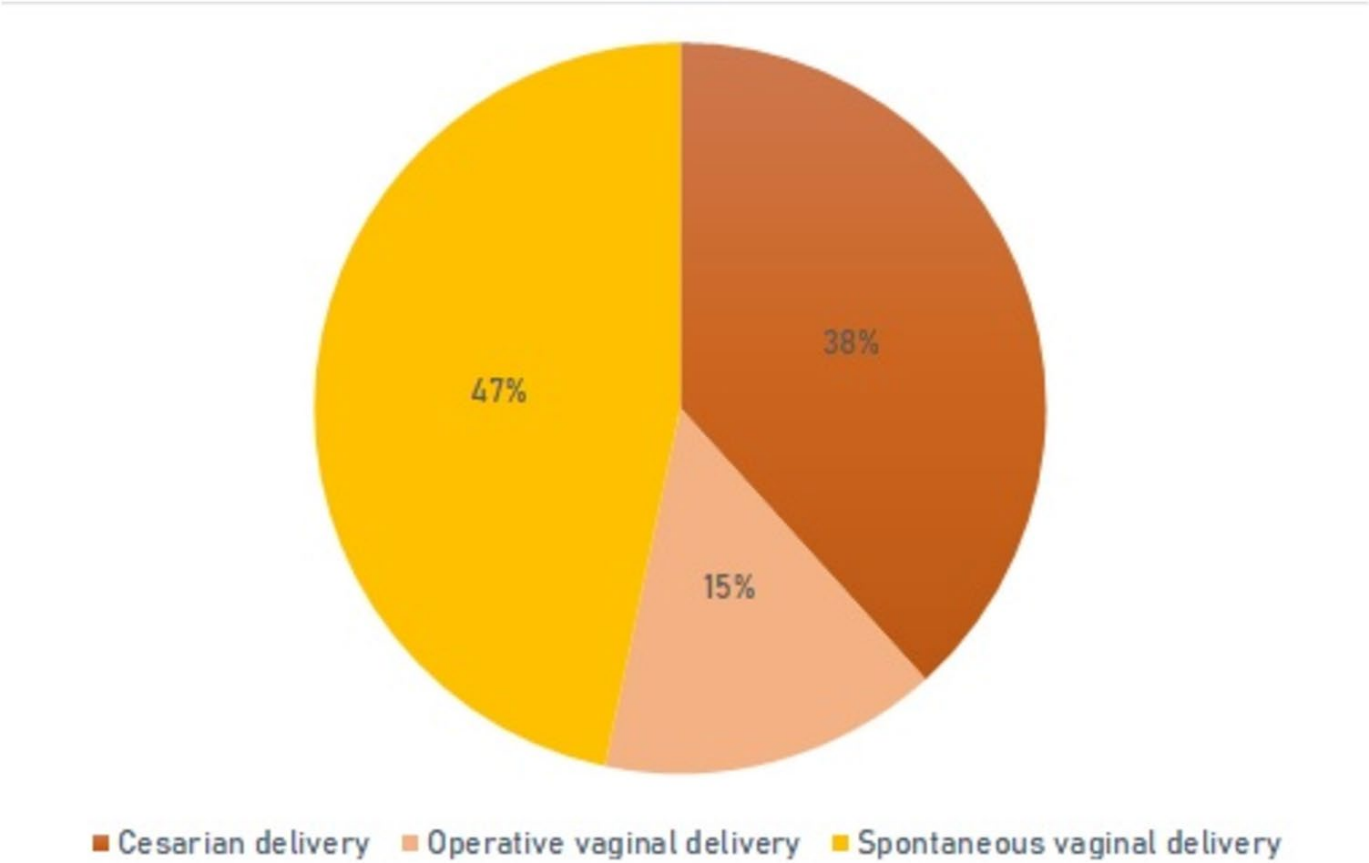
Mode of delivery

Overall, during 40 (63%) pregnancies, women experienced a worsening of myasthenic symptoms. The highest rate was seen in the post-partum period (40%), followed by the first trimester (33%), rate of myasthenic worsening was lowest in the second and third trimester (Fig. 1). Increase in MG-specific medication was necessary in 13 (19.1%) patients, MG-specific medication could be decreased in 22 (32.2%), in 33 (48.5%) of patients MG-specific medication remained unchanged (Fig. 2). Escalation therapy was exclusively necessary in the post-partum period with 9 patients (14.1%) being treated with IVIg and one patient (1.6%) requiring PLEX. Rate of mode of delivery was 46.7% for spontaneous vaginal birth, 38.3% for C-section and 15% for operative vaginal delivery (Fig. 3).

### Neonatal outcome

Mean week of pregnancy at delivery was 38.8 weeks, rate of premature deliveries was 13%. Mean weight at birth was 3111 g, rate of SGA was 16%. Mean APGAR score at 1, 5 and 10 min was 8.0, 9.2 and 9.6, respectively. Rate of TNMG as well as AMC was 3% (two cases each, Table 2).

**Table 2:** Neonatal outcome

Characteristic	
Week of pregnancy at delivery, mean (SD)	38.8 (2.4)
Prematurity, *n* (%)	7 (13)
APGAR score, mean (SD)	
At 1 min	8.0 (2.1)
At 5 min	9.2 (1.3)
At 10 min	9.6 (0.8)
Weight at birth, g, mean (SD)	3111 (703)
Small for gestational age, *n* (%)	9 (16)
Transient neonatal MG, *n* (%)	2 (3)
Arthrogryposis multiplex congenita, *n* (%)	2 (3)

## Discussion

### Principal findings

Our findings confirm that risk for worsening of myasthenic symptoms is the highest in the first trimester and post-partum period, and that rate of C-section in MG patients giving birth is not increased. However, overall data on mode of delivery suggest a slightly increased rate of operational vaginal birth in MG patients. Neonatal outcome was favourable in our cohort with a low rate of TNMG and AMC/FARAD.

## Results

Overall rate of worsening of myasthenic symptoms was 63%. This is above the overall rate of 33.4% reported in a recent review [5]. However, it has to be stated that most publications use the term “exacerbation” without providing a definition which gives way to different interpretations [6, 7]. Criteria of myasthenic exacerbation as defined by the German guidelines were fulfilled in 10 cases and exclusively in the post-partum period, requiring treatment with IVIg and PLEX in 9 (14.1%) and 1 (1.6%) patients, respectively. There was no case of myasthenic crisis defined as necessity for intubation and mechanical ventilation [17]. In our study, we refer to symptom aggravation not fulfilling the criteria of exacerbation as “worsening”.

Rate of C-section was 38.3% in our cohort. Mode of delivery in MG patients shows great variability across studies that might be explained by geographical and temporal disparities as well as variances in the level of care provided by the hospitals where birth was given [5, 7, 18]. A recent nationwide Swedish register-based cohort study found higher elective caesarean section rate in MG patients compared to matched controls [19]. The overall C-section rate in Germany is 30.9% [20]. Data analysed in this study stem from patients treated at a university hospital of maximum care with over 5000 births per year with a higher percentage of high-risk pregnancies compared to basic care hospitals. Overall rate of C-sections at Charité – Universitätsmedizin Berlin was 40.2% in the first six months of 2023. The higher overall C-section rate at our centre is in line with the status as a tertiary care centre with a large referral radius for high-risk pregnancies and therefore comparable to our cohort of MG patients. A recent review on pregnancy in MG found that the vast majority of C-sections in MG patients is performed for obstetric reasons and only a small percentage with MG as indication [5]. Unfortunately, our data did not allow for a clear distinction between obstetric vs. MG indication for C-section, we therefore opted to exclude this item in our analysis. The contractile apparatus of the uterus consists of smooth muscle cells and is therefore not affected of myasthenic symptoms. However, during expulsion stage, striated muscles are needed. The slightly higher rate of operational vaginal birth of 15% in our cohort compared to the overall rate at our hospital (9.7% in the first six months of 2023) might be an indicator for prematurely fatiguing striated musculature in MG patients during expulsion stage.

We had two cases of AMC in the same patient in our cohort. Before being treated at our centre, the patient had a history of an abortion with hydrops fetalis and intrauterine death of the foetus at 22 weeks of gestation (WOG). In her second pregnancy, she presented with sonographic signs of AMC/FARAD at 26 WOG. The presence of AChR-ab was confirmed and immunomodulatory treatment with PLEX and IVIg was initiated at our centre but the fetus died at 30 WOG. In her third pregnancy, we initiated treatment with PLEX and IVIg at 23 WOG after again detecting sonographic signs of AMC/FARAD. After premature rupture of membranes, a C-section was performed in 30 WOG with death of the neonate directly after birth. AMC is a descriptive term for conditions with multiple congenital contractures and has a very heterogenous aetiology with more than 400 disorders presenting with AMC identified to date [21]. In MG, the pathogenesis of AMC and the related FARAD is a transplacental transfer of maternal autoantibodies that selectively or primarily inhibit the fetal subunit of the acetylcholine receptor during a critical period of fetal muscle development leading to lack of intrauterine fetal movements [13, 14]. The clinical picture of AMC is a spectrum that ranges from joint contractures to intrauterine or neonatal death due to pulmonary hypoplasia and hydramnios [22]. Mothers can be asymptomatic and the condition usually recurs in each pregnancy as was the case in our cohort [23]. Children with AMC can only be offered supportive treatment. The main therapeutic target are mothers with a respective history to be treated with immunosuppressive/immunomodulatory treatment during pregnancy including intravenous immunoglobulins and plasma exchange [11].

TNMG is also caused by a transplacental transfer of maternal IgG antibodies but at a later stage and not with predominant inhibition of the fetal subunit of the AChR. Rate of TNMG was low in our cohort with 3%. A recent review found that the incidence of TNMG is 13% [5]. There were two TNMG cases from two different mothers. TNMG can affect newborns of MG mothers with all autoantibody constellations [11, 24, 25] and does not seem to correlate with antibody titre or maternal disease severity. However, thymectomy prior to pregnancy and longer disease duration might be protective factors [7]. In our cohort, TNMG occurred in newborns from one AChR-ab-positive (no thymectomy) and one seronegative mother (thymectomy performed prior to pregnancy with histopathological picture of thymus hyperplasia). Rate of thymectomy in our cohort was fairly high with 60%. Moreover, mean disease duration at delivery was 6 years in our cohort. It is known that MG disease activity is the highest during the first 2–3 years after disease onset. At our clinic, we advise female patients with desire to have children, if possible, to wait until disease control or remission is achieved before becoming pregnant [26]. These factors might partially explain the low rate of TNMG in our cohort.

### Clinical implications

Maternal and neonatal outcome in our cohort was favourable with a low rate of myasthenic exacerbations requiring escalation therapies as well as a low rate of TNMG and AMC/FARAD. However, due to the highest risk of myasthenic exacerbations in the post-partum period and onset of TNMG during the first 24 h after delivery, pregnant MG patients should be advised to deliver in hospitals with collaborating departments of obstetrics, neonatology and neurology and mothers as well as neonates should be closely monitored. Cases of maternal autoantibody induced AMC/FARAD are rare; however, pregnant MG patients should receive repeated ultrasound scans to detect early symptoms. On the other hand, in case of AMC/FARAD signs in pregnancies overall, fetal medicine specialists should be aware of the potential differential diagnosis of AChR-ab as an underlying cause.

### Research implications

To the best of our knowledge, there is no prospective study on maternal and neonatal outcome in MG patients to date. A prospective pregnancy registry, preferably on a European or international level, would be desirable to better assess risk and protective factors of maternal and neonatal outcome in the orphan disease MG.

### Strengths and limitations

The retrospective design of this study inherently carries a lower level of evidence, the possibility of recall bias in the patients that answered the questionnaire and less detailed information compared to prospective studies. This also includes lack of MG-specific outcome scores used in clinical trials, such as MG activities of daily living profile (MG-ADL) and Quantitative MG score (QMG) [27, 28]. Clinical worsening of myasthenic symptoms was based on neurological examination documented in the medical record. The limited number of events did not allow for an analysis of clinical factors (e.g. antibody status, type of MG-specific medication) potentially associated with maternal and neonatal outcome. However, our study adds to the scarce literature on this subject with a relatively large sample size of our descriptive cohort and the first German experience on this subject to date.

## Conclusions

Maternal and neonatal outcome in MG patients is favourable. In the light of a study showing that approximately half of female MG patients abstained from having (further) children due to their disease [4], our data might help neurologists and obstetricians to advise and support MG patients who wish to have children and to do so under the safest conditions possible.

## Data Availability

Data not provided in the article because of space limitations may be shared (anonymized) at the request of any qualified investigator for purposes of replicating procedures and results.
